# The Impact of CRISPR/Cas9 Technology on Cardiac Research: From Disease Modelling to Therapeutic Approaches

**DOI:** 10.1155/2017/8960236

**Published:** 2017-12-25

**Authors:** Benedetta M. Motta, Peter P. Pramstaller, Andrew A. Hicks, Alessandra Rossini

**Affiliations:** Institute for Biomedicine, Eurac Research, Affiliated Institute of the University of Lübeck, Bolzano, Italy

## Abstract

Genome-editing technology has emerged as a powerful method that enables the generation of genetically modified cells and organisms necessary to elucidate gene function and mechanisms of human diseases. The clustered regularly interspaced short palindromic repeats- (CRISPR-) associated 9 (Cas9) system has rapidly become one of the most popular approaches for genome editing in basic biomedical research over recent years because of its simplicity and adaptability. CRISPR/Cas9 genome editing has been used to correct DNA mutations ranging from a single base pair to large deletions in both *in vitro* and *in vivo* model systems. CRISPR/Cas9 has been used to increase the understanding of many aspects of cardiovascular disorders, including lipid metabolism, electrophysiology and genetic inheritance. The CRISPR/Cas9 technology has been proven to be effective in creating gene knockout (KO) or knockin in human cells and is particularly useful for editing induced pluripotent stem cells (iPSCs). Despite these progresses, some biological, technical, and ethical issues are limiting the therapeutic potential of genome editing in cardiovascular diseases. This review will focus on various applications of CRISPR/Cas9 genome editing in the cardiovascular field, for both disease research and the prospect of *in vivo* genome-editing therapies in the future.

## 1. Introduction

The possibility to perform gene therapy, correcting specific disease-causing mutations in the germline, has emerged more than 40 years ago [1]. In the 1980s, with an increased understanding of the transduction capabilities of retroviruses, scientists have investigated the clinical potentiality of these viruses for gene therapy applications. It has been demonstrated that the retroviruses could correct phenotypes in cells from diseased patients [2–4]. However, the use of retroviruses has faced some important impediments, like their instability, toxicity, and inability to infect nondividing cells. For these reasons, other viral and nonviral gene delivery tools have been developed, which include adeno-associated virus (AAV) vectors, herpes vectors, and liposomes [5, 6]. The first clinical trial of cardiovascular gene therapy was the delivery of the gene encoding for the vascular endothelial growth factor (VEGF) into patients with peripheral arterial disease, but it was not considered an ideal treatment as many patients presented lesions not amenable to gene delivery [7]. In the first decade of this century, efforts have focused on the development of efficient and safe tools to deliver genes into patient cells. Through mutagenesis with insertion and substitution, reengineered AAV vectors that are both safer and well tolerated have been developed [8]. Recently, genome-editing technology has emerged as a new powerful method to elucidate gene function and to correct human disease-causing variants, enabling the production of genetically modified cells and organisms. The main features of genome editing tools is represented by the fact that they rely on nucleases introducing DNA double-strand breaks (DSBs) to induce gene mutation by stimulating endogenous repair mechanisms [9]. The most critical part consists in the introduction of the DSBs at the specific desired target site. This specificity of action is addressed differently by different tools, the most commonly used being zinc finger nucleases (ZFNs), transcription activator-like effector nucleases (TALENs), and clustered regularly interspaced short palindromic repeats- (CRISPR-) associated 9 (Cas9) systems [10, 11]. Of these, the CRISPR/Cas9 system, which was firstly described in 2012 by the groups of Jinek et al. [12], has brought considerable progress to the field. This system is sufficiently easy and efficient to edit genes in virtually any organism and cell type [13, 14] and, because of its simplicity and adaptability, CRISPR/Cas9 has rapidly become one of the most popular approaches for genome editing [15–18].

Within the context of basic understanding of disease molecular mechanisms and disease treatment, the CRISPR/Cas9 system has just started to reveal its potential, appearing as a tremendously powerful tool for basic research applications, such as functional interrogation of new loci and generation of novel animal models, as well as for novel clinical approaches [15, 17, 19–21]. Currently, clinical trials using CRISPR/Ca9-edited human cells are ongoing, for example, to treat cancer [22, 23].

Cardiovascular diseases are still a major health problem with increasing prevalence [24, 25], and a remaining challenge is to gain a deeper knowledge of the mechanisms behind the development of both common and less common causes of cardiovascular mortality and morbidity. Cardiovascular diseases include several medical conditions involving the blood vessels and the heart. These are mainly coronary artery disease (CAD), cerebrovascular disease (stroke), peripheral artery disease (PAD), cardiomyopathies, rheumatic heart disease, arrhythmias, hypertensive heart disease, and congenital heart diseases [26]. Thanks to genetic testing and bioinformatics analyses, we are increasingly able to identify subjects susceptible to particular cardiac diseases. However, further mechanistic studies aimed at understanding the cause of the diseases are limited by the fact that the isolation and culture of primary human cardiomyocytes for cardiovascular research is extremely difficult [27]. In this context, researchers all over the world have invested time and resources to ameliorate animal and cell models for cardiac diseases. The CRISPR/Cas9 technology has been used to generate mouse models of genetic diseases, such as severe cardiomyopathy, in a shorter time than with the traditional homologous recombination technique [28–30]. Additionally, it is now possible to inject CRISPR/Cas9 into cell embryos in rats, rabbits, and primates, which are used to study cardiovascular diseases [31, 32]. With the advent of induced pluripotent stem cell (iPSC) technology, the researchers have also been able to generate alternative cell models to investigate the molecular mechanisms of the diseases. In this context, the CRISPR/Cas9 technology provides a straightforwarc mechanism to elucidate how the cells misbehave by allowing the reversion of the causal mutation [33–35]. Finally, in the cardiovascular field, important steps have been made towards therapeutic applications [34, 36–38].

The present review will focus on the various applications of the CRISPR/Cas9 genome editing tool in the cardiac field, ranging from its application to human and animal models to its therapeutic potential, describing the advantages and potential disadvantages of the system.

## 2. The CRISPR/Cas9 System

The CRISPR/Cas9 technology derives from the adaptive immunity of the bacterium *Streptococcus pyogenes* [12]. The Cas9 nuclease mediates anti-phage activity thanks to its combination with the clustered regularly interspaced short palindromic repeats (CRISPR) loci. These loci are short, repetitive sequences consisting of 30–40 bp and intercalated with spacer sequences matching virus genomes [39]. The CRISPR loci are transcribed into a long RNA which is subsequently cleaved by the CRISPR-associated endoribonucleases (Cas) to release small CRISPR RNAs (crRNAs). These crRNAs then form a Cas-RNA complex that recognizes the genome of the virus and proceeds to cleave it.

In its modified form for genome editing, the CRISPR/Cas9 system consists of a RNA guide (gRNA) sequence to target the nuclease Cas9 to a specific site in the genome. The gRNA is composed of a short RNA sequence necessary for the binding of Cas9 plus a ~20 nucleotide sequence, called the protospacer, which defines the DNA target to be modified [12]. The Cas9 nuclease contains an HNH-nuclease and the RuvC-like nuclease domains. It first recognizes a conserved sequence named protospacer-adjacent motif (PAM), which flanks the target DNA [40, 41]. After binding the PAM, Cas9, by its HNH and RuvC catalytic domains, generates a blunt double-strand break (DSB) that can be repaired by either nonhomologous end joining (NHEJ) or a homology-directed repair (HDR) system [10, 12, 42]. NHEJ repair predominantly occurs during G1 phase, while HDR is most prominent during S and G2 phases. NHEJ does not require a repair template or extensive DNA synthesis and it is faster than HDR in repairing DSB. Occasionally, it introduces small insertions or deletions (indel) at the cleavage site. If the indel occurs within the coding sequence of a gene, it leads to a frameshift mutation, which can result in a gene knockout [43], making it unsuitable for gene-correction purposes. In contrast, HDR uses a DNA template, or the nonmutant homologous chromosome, to achieve a high-fidelity repair and can be used to introduce a precise mutation or insertion by recombination [44–47]. The efficiency of HDR in repairing DSB is relatively low [17] and less frequent compared to NHEJ in proliferating human cells [48]. The efficiency of HDR can be promoted by transient inhibition of NHEJ [49, 50].

Of note, the presence of a limited number of PAM sites within the eukaryotic genome [12, 51] have represented a limitation for the precision of the initial genome editing applications [52]. To overcome this problem, the number of targetable sites within the genome has been increased thanks to the characterization of the CRISPR/Cas system in different bacterial species with different PAM sites [15, 53]. Additionally, Kleinstiver et al. [54] have demonstrated that the commonly used *Streptococcus pyogenes* Cas9 can be modified to exhibit altered PAM specificity, thus recognizing alternative PAM sites throughout the genome of human and model organisms. Several studies have taken advantage of the HDR repair machinery to introduce precise single point mutations or knockins in a target gene providing a homologous repair template. The NHEJ repair machinery is preferred as a way to introduce insertions and/or deletions which can disrupt the targeted locus [55, 56]. It is also possible to generate large deletions or genomic rearrangements, such as inversions or translocations, using a pair of gRNA-directed Cas9 nucleases [57]. The CRISPR/Cas9 system has many other applications other than gene editing, such as the regulation of endogenous gene expression. A nuclease-deficient Cas9, called “dead Cas9” (dCas9), has been developed to create inactive fusion proteins capable of targeting either the promoter or regulatory sequences of a gene to alter its expression by interfering with the normal transcriptional machinery [56, 58]. Coupling of dCas9 to a transcriptional repressor like KRAB (CRISPRi) can reduce the transcription of endogenous human genes. This approach was described to consistently reduce gene expression in eukaryotic models [58]. More recently, it has been demonstrated to repress gene expression also in bacteria and human induced pluripotent stem cells [59, 60]. In the same way, fusing dCas9 with a transcriptional activation domain like VP64 or p65 (CRISPRa) can increase the expression of endogenous human genes [61]. CRISPRa enables also a multiplexed genes activation, using single gRNAs (sgRNA) targeting more genes simultaneously [62]. dCas9 can be also fused to functional domains of DNA methylation or demethylation enzymes, or histone modifiers for epigenome-editing [63, 64]. Different Cas9 nucleases have been generated to modify nuclease activity or binding selectivity. The Cas9D10A, mutant CRISPR Nickase, has been produced to selectively make a single-strand DNA cut at the target sequence. By this double-nicking approach, the Cas9D10A creates “sticky ends” which ensure that the DNA fragment is inserted into the genome in the right orientation [65, 66]. The CRISPR/Cas9 system has been also used for live-cell labelling, coupling dCas9 to fluorescent proteins to visualize specific genomic loci [67].

## 3. The CRISPR/Cas9 System in Human Cell Models of Cardiac Disease

The introduction of genome editing has revolutionized basic and translational research together with the innovative discovery of induced pluripotent stem cells [68].

Due to their close similarity to embryonic stem cells (ESCs) [68, 69] and to the fact that they are not burdened by the same ethical concerns, iPSCs are currently the most suitable model to study cardiomyogenesis on human cells [70]. Further, iPSCs are a virtually infinite source of human cardiomyocytes and represent an invaluable tool in the cardiac field, notably lacking human *in vitro* models [71]. Given that iPSC-derived cardiomyocytes (iPSC-CMs) show a phenotype still far from that of adult human cardiomyocytes [72, 73], a lot of effort has been made by the scientific community to develop protocols able to ameliorate their *in vitro* maturation in order to generate better models of cardiac pathologies [74]. Up to now, several cardiac diseases have been investigated using iPSC-CM, which have been shown to be a good model for inherited arrhythmogenic disorders [75–78]. Patient-specific iPSCs have been generated from individuals of a family affected by long-QT syndrome type 1 and induced to differentiate into functional cardiac myocytes. These cells exhibited prolongation of the action potential, altered delayed rectifier potassium current (IK) channel activation and deactivation, and an abnormal response to catecholamine, which are the electrophysiological features of the disorder [76]. Accordingly, iPSC-CMs derived from a catecholaminergic polymorphic ventricular tachycardia (CPVT) patient demonstrated anomalous electrophysiological features, including delayed afterdepolarizations [79]. In addition, iPSC-CMs have been generated to study the single-cell alterations in structural cardiac defects such as those found in dilated cardiomyopathy (DCM) and hypertrophic cardiomyopathy (HCM). Sun et al. generated cardiomyocytes from iPSCs of DCM patients carrying a mutation in the gene encoding for the sarcomeric protein cardiac troponin T and showed that iPSC-CMs recapitulate the morphological and functional phenotypes of affected hearts, such as altered Ca^2+^ handling, a decreased contractility, and abnormal distribution of sarcomeric *α*-actinin [80]. Further, iPSCs from an HCM patient with a single missense mutation in the MYH7 gene exhibited disorganized sarcomeres, electrophysiological irregularities, and an increase of genes involved in cell proliferation [81].

It is therefore evident that the parallel advances in genome editing and iPSC technology is now offering the opportunity to investigate the pathophysiological mechanisms of inherited cardiac diseases directly in human cell models [82–84]. Here, the CRISPR/Cas9 tool allows for a relatively efficient and easy generation of isogenic cell lines differing only by the DNA sequence of interest [85], thus eliminating other confounders like genetic background and epigenetic memory [35, 85]. Until now, the CRISPR/Cas9 system has already been proven to be an effective and useful approach to create gene KO or knockin in human cells [15–17] and in particular in iPSCs [86, 87]. Moreover, this system has the potential to correct genetic mutations in iPSC-related disease models [88–90] and has already started to be applied to the study of different cardiac diseases, such as Barth syndrome, an X-linked genetic heart disease [91]. Specifically, Wang and coworkers have recently given an excellent example of the potential that combined CRISPR/Cas9 and iPSC technology provides. They generated iPSCs from patients with Barth syndrome and characterized mitochondrial CM abnormalities associated with this pathology. By introducing a mutation in the tafazzin (*TAZ*) gene in iPSCs from healthy donors through Cas9-mediated genome editing, they also demonstrated a causal relationship between the *TAZ* gene mutation and the mitochondrial phenotype. Importantly, the administration to Barth syndrome-derived iPSC-CMs of the antioxidant mitoTEMPO was efficient in suppressing the excess of mitochondrial ROS production and led to the normalization of sarcomere organization and contractility [91]. Recently, the CRISPR/Cas9 system has been also used to evaluate the pathogenicity of titin gene mutations in dilated cardiomyopathy. Missense or frameshift titin mutations have been introduced in iPSCs and contractile deficits have been subsequently evaluated in iPSC-CM [33]. Additionally, iPSC-CMs have been generated from two independent groups of patients with Jervell and Lange-Nielsen syndrome (JLNS), which is one of the most severe of the heart rhythm cardiac arrhythmias [92]. The cardiomyocytes showed the typical features of JLNS, including action potential prolongation and a severe reduction or absence of IKs [35]. More recently, Yamamoto et al. established a disease-specific iPSC clone from an individual with Long QT syndrome (LQTS) carrying a heterozygous *CALM2* mutation as an *in vitro* disease model, reproducing the disease phenotype. Additionally, they excised the mutant allele using the CRISPR/Cas9 system and rescued the abnormal electrophysiological properties [93]. Importantly, CRISPR/Cas9 can be also used to introduce DNA changes in noncoding regions. This approach was successfully used in human iPSCs to delete the sequence adjacent an intronic single nucleotide polymorphism in the *PHACTR1* gene, strongly associated with premature myocardial infarction [94].

## 4. The CRISPR/Cas9 in Animal Models of Cardiac Diseases

The experimental induction of specific mutations into the genome of model organisms put the basis for our current understanding of cardiac function [95, 96]. However, classical tools show significant limitation: chemical mutagenesis completely lacks specificity, while gene targeting by homologous recombination is complex and time consuming [97]. In this context, the development of CRISPR/Cas9 technology has represented a powerful breakthrough for the generation of different animal models to the aim of heart disease investigation.

In the present paragraph, we are reviewing current findings and novel approaches in three different organisms widely and successfully used to study cardiac diseases: *Drosophila melanogaster*, zebrafish (*Danio rerio*), and mouse (*Mus musculus*).

The fruit fly (*Drosophila melanogaster*) has been used as model organism in biomedical research to study a broad range biological processes for over a century, including, among the others, genetics, inheritance, and development [98]. The success of flies over vertebrate models relies mainly on the fact that they are cheap, have a short life span, and produce a huge number of embryos. Although the *Drosophila* heart is a linear tube, thus very different from the 4 chambers of a mammalian heart, it is reminiscent of the vertebrate heart tube and exhibits evolutionarily conserved elements related to both heart development and function [99]. Recently, an RNAi-based screening has been successfully conducted in *Drosophila* to identify critical pathways for cardiovascular homeostasis, which led to the identification of the CCR4-Not complex as an important new player in cardiac function, that was confirmed in mice and humans [100]. Therefore, genome-wide screens in flies have demonstrated their potential to identify conserved candidate genes involved in cardiac function. However, classic RNAi screens suffer from important drawbacks, namely, (i) RNAi is successful only when the target is actively transcribed [101], (ii) it often leads to only partial protein depletion [102], and (iii) it allows for the functional evaluation of coding region, thus excluding the interrogation of regulatory and intergenic elements [103]. In this context, the CRISPR/Cas9 technology represent a substantial improvement with respect to all the abovementioned limitations of conventional approaches. Of note, CRISPR/Cas9-mediated mutagenesis has been successfully reported in *Drosophila* [104–106]. Recently, Port and colleagues [107] achieved biallelic targeting of genes in selected somatic cells, demonstrating the feasibility of restricting CRISPR/Cas9-mediated mutagenesis in time and space and thus paving the way for future high-throughput genetic screening in *Drosophila* heart.

The zebrafish has gained a wider and wider popularity as model for cardiac development and disease mainly because of its relative ease of use for genetic analyses in comparison to mouse. Further, thanks to the fact that the need for active oxygen delivery is very limited during the first week of development [108], the use of zebrafish model allows for the investigation of cardiovascular defects causing embryonic death in other organisms. Intriguingly, the zebrafish heart exhibits a strong regenerative potential that is lacking in other vertebrates such as rodents [109, 110]. This aspect has exponentially increased the interest for the zebrafish model, with the aim of understanding the molecular basis of cardiomyocyte regeneration which might be eventually applied to humans. Although composed only of two chambers, the zebrafish heart possesses electrical features that resemble the human heart. For instance, it displays a pacemaker, sinoatrial node-like region [111], and the ventricular cardiomyocytes exhibit action potential features resembling those of the human heart [112, 113]. Zebrafish models have been created not only to investigate the molecular determinants of cardiac development [114] but also to gain deeper insights into cardiac diseases such as inherited cardiomyopathies [115]. Noteworthy, knockdown models generated by the standard morpholino method show discrepancy when compared to genome-editing based approach [116]. This observation underlines again the importance of carrying out gene-deficiency studies using genome editing instead of RNA based tools [117]. Until now, several CRISPR/Cas9 based systems have been reported for genome editing in zebrafish [118, 119]. Recently, the group of Ablain et al. [120] has reported a tool to generate tissue-specific gene knockout by injecting guide RNA and Cas9 mRNA into the one cell-stage embryo. As already described in *Drosophila*, this opens the possibility to control the disruption of a specific gene only in cardiac cells. This greatly improves the potential of loss-of-function screens in zebrafish, which can be used in the near future not only for characterizing cardiac phenotypes in embryos but also for modelling adult cardiac diseases [121].

Being applicable directly to embryos, the CRISPR/Cas9 system has become the most popular tool for mouse engineering. Older approaches required the induction of homologous recombination in mouse embryonic stem cells, the selection of mutated ESCs through antibiotic resistance, the excision of the antibiotic cassette, and the injection of ESCs into the blastocyst recipient mice [28]. On the contrary, CRISPR/Cas9-based technology allows for the generation of mutated mice in just one step, consisting of the coinjection into the zygotes of Cas9 mRNA, different sgRNA, and DNA donors [29]. Using this approach, Kaneko and others have shown that phospholamban knockout improves cardiac function in a mouse model of heart failure induced by calsequestrin overexpression [122]. Further, it has been proven that CRISPR/Cas9 genome editing applied to mouse zygotes can correct a Duchenne muscular dystrophy-causing mutation, restoring dystrophin expression not only in skeletal muscle but also in the cardiac tissue of mdx mice [123].

The CRISPR/Cas9 system has further shown its tremendous potential when it was demonstrated that it can correct genetic defects in postnatal/adult mice [124]. Ding et al. used the CRISPR/Cas9 system to edit genes in somatic cells *in vivo*. They disrupted the proprotein convertase subtilisin/kexin type 9 (*PCSK9*) gene, leading to reduced blood cholesterol levels, lowering the risk of developing coronary heart disease (CHD) due to higher LDL cholesterol levels [13]. This study demonstrated the therapeutic potential of CRISPR/Cas9 tool in preventing CHD. Notably, one of the major issues with the CRISPR/Cas9 system is related to the molecular size of the CRISPR/Cas9 components, which are not easily packaged into adeno-associated virus (AAV) for *in vivo* delivery [36]. To overcome this limitation, different strategies have been used, like a dual-vector approach that allows for a finer control of the ratio between administered nuclease versus targeting elements components [125]. Additionally, transgenic mice expressing Cas9 only in cardiomyocytes have been produced by the group of Caroll et al. [30]. As a proof of concept, the investigators reported that the ablation of Myh6 mediated by the delivery of sgRNA against Myh6 through AAV resulted in mice with impaired cardiac performance and significant hypertrophy. This elegant work describes an efficient and relatively easy cardioediting tool to explore the specific role in cardiac function and/or development of genes whose expression is not limited to the heart.

For the sake of completeness, it is also important to note that the CRISPR/Cas9 system has been successfully used to target the embryo of other mammals such as rats [32] and monkeys [31]. Intriguingly, Niu and colleagues have demonstrated the feasibility to specifically target multiple genomic sites in a one-step mutagenesis approach applied to one-cell monkey embryos [31]. It is well recognized that mouse, although being the most used mammals for genetic studies, presents several limitations when used to model human electrophysiological disorders [126], dyslipidemia [127], and myocardial infarction [128]. The relative simplicity of the CRISPR/Cas9 technology and the proof of concept of its feasibility in a wide range of organisms [15–17, 104, 105, 129, 130] are therefore now enriching the scientific community with the possibility to use *in vivo* models that are more representative of human cardiac physiology such as rabbits and monkeys.

In summary, the CRISPR/Cas9 technology appears to be one of the most efficient tools for genome editing of a wide range of animal models, both at the stage of one-cell embryo and in postnatal life. The continuous evolution of bioinformatics tools to improve the design of guide RNAs [131], along with the constant optimization of experimental conditions, have enabled the establishment of very consistent protocols that are completely reliable for the generation of knockout and knockin point mutations useful for cardiac disease modeling, cardiac gene editing, and for exploration of potential gene therapy [132, 133].

## 5. The Therapeutic Potential of the CRISPR/Cas9 System

In the recent years, mainly thanks to next-generation sequencing approaches [134], we have witnessed a constant increase in human genetic information regarding not only monogenic but also complex diseases [135]. These advances, together with the growing evidence for the role of common variants in disease predisposition [136, 137], have enabled the search for application of genome-editing strategies to the treatment of complex disorders, including cardiovascular diseases.

In theory, the CRISPR/Cas9 system can be used for therapy not only by correcting disease-causing mutations but also introducing protective mutations and targeting viral genomes [138].

Recently, Limpitikul et al. managed to correct a mutation in the calmodulin 2 (*CALM2*) gene in iPSC-CM, leading to a functional rescue of long QT syndrome-triggered cardiac events [139]. LQTS-associated calmodulinopathies are due to a mutation occurring in one of the 3 calmodulin genes, *CALM1*, *CALM2*, and *CALM3.* In this work, the proband affected by the malignant calmodulinopathic long QT syndrome harbored a mutation in only 1 of 6 redundant calmodulin-encoding alleles. Here, the authors used a CRISPR interference (CRISPRi) strategy to selectively correct the mutated allele alone. CRISPRi technology uses the dCas9 protein to regulate gene expression at the transcriptional level. The dCas9-sgRNA complex binds to the target gene at the promoter or coding sequence and it impedes the activity of RNA polymerase, preventing transcription initiation or elongation [140]. By successfully inhibiting just the mutant allele, the authors successfully demonstrated an approach that is applicable in principle not only to other calmodulinipathies, such as catecholaminergic polymorphic ventricular tachycardia, but also to a variety of cardiac and noncardiac diseases characterized by a redundancy of the affected gene.

**Figure 1:**
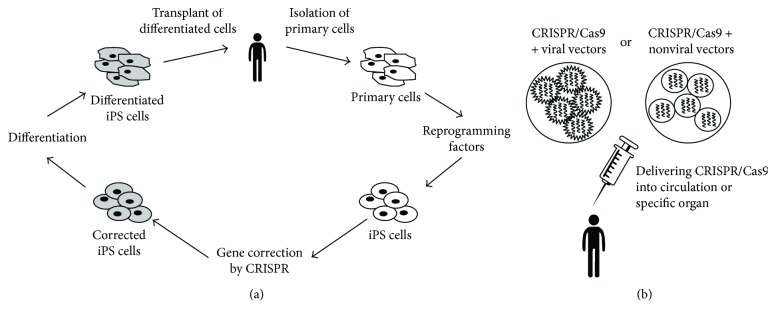
Therapeutic approaches by genome editing. There are two possible approaches of genome editing in cardiovascular diseases. (a) Somatic cells can be isolated from patients and reprogrammed in iPS cells. The patient-specific iPSCs can be modified ex vivo, and after the editing they can be differentiated and transplanted back into the patient. (b) The mutations can be directly edited *in vivo*, delivering the complex CRISPR/Cas9 system-delivery tool at the desired genomic site in the specific tissue.

It is important to note that, although attractive as a strategy, the efficacy of genome-editing approaches for therapy relies on the development of a successful delivery strategy of the CRISPR/Cas9 system into patients. This could be achieved either ex vivo, using a “patient-to-patient” strategy by modifying autologous cells and then transplanting them back into the patient, or *in vivo*, by direct injection of the CRISPR/Cas9 system into the patient (Figures 1(a) and 1(b)).

In the first case, the iPSCs obtained from a patient can be edited for the correction of a specific mutation or can be made resistant against disease. The edited iPSCs can be differentiated into the desired cell type (such as cardiomyocytes) and transplanted back into the patient. However, this approach seems to be limited to systems where cells engraft efficiently after reinjection. Unfortunately, the efficiency of stem cell homing and engraftment into the damaged heart is known for being extremely low [141, 142]; therefore, it still represents a major challenge for the possibility of ex vivo editing of autologous iPSC-derived cardiomyocytes. Promisingly, Chong et al. recently generated cardiomyocytes from embryonic stem cells able to successfully engraft and repair the injured myocardium in a primate model of myocardial infarction [143]. However, especially in the case of inherited diseases, the number of corrected cells might remain too low compared to the volume of the host tissue. Thus, *in vivo* therapeutic genome editing in the cardiovascular field has to deal with different barriers [144, 145]. More improvements in maturation of iPSC-CMs as well as the generation of organoids or 3-dimensional structures will be necessary to fully realize the utility of this technique.

It seems therefore reasonable that for genetic cardiac diseases, the *in vivo* editing approach involving the direct injection of the nuclease system might be a better method. In fact, *in vivo* delivery of the CRISPR/Cas9 tool can be seen as a perspective for the correction of mutations in inherited cardiac disorders such as long QT syndrome [146]. A similar approach has been used to correct a dominant mutation in the CRYGC gene that leads to cataracts in mouse [147].

An example of *in vivo* disruption of a gene as a therapeutic approach is provided by the targeting using CRISPR/Cas9 of the PCSK9 gene in a “humanized” mouse model, which reduced cholesterol levels and consequently the risk of myocardial infarction [148]. CRISPR/Cas9 editing has also been successfully used to treat tyrosinemia and prevent cardiovascular diseases in *in vivo* adult mice [149].

As we have seen, CRISPR/Cas9 has been used to correct disease-causing DNA mutations ranging from a single base pair to large deletions. To correct the altered function of large proteins, it is possible to use a multiplexed CRISPR/Cas9 with two gRNAs adjacent to the DNA mutation to be deleted and to restore the functional protein. This process has been tried for the treatment of Duchenne muscular dystrophy [150].

As a final remark, it should be noted that the CRISPR/CAS9 system can lead to the targeting of multiple tissues, which can be seen as both a great potential in the case of diseases targeting multiple organs, and conversely as a great limitation when a selective tropism for a specific tissue is desired but difficult to achieve. In fact, the majority of *in vivo* studies deliver the CRISPR-Cas9 components via AAVs, but the tissue tropism of AAVs is restricted to a few organs [151].

## 6. Advantages and Disadvantages of the CRISPR/Cas9 System

As outlined in the previous chapters and summarized in Table 1, the CRISPR/Cas9 system has many advantages over other existing technologies. The Cas9 enzyme is guided to a specific DNA sequence by a single guide RNA (gRNA) that can be easily cloned. In contrast, ZFNs and TALENs are fusion proteins of designed DNA-binding sequences and the Fok I nuclease cleavage domain. Because the Fok I domain needs to dimerize to be active, two proteins are required for genome editing experiments, compared with the single gRNA of CRISPR/Cas9 system [152, 153]. Using multiple gRNAs, it is possible to target multiple genomic loci simultaneously introducing mutations in several genes [15, 17, 154]. CRISPR/Cas9 has been demonstrated to be effective for editing different human cells [15–17]. Moreover, the CRISPR/Cas9 can be used to screen DNA noncoding regions to identify regulatory elements to understand how genetic variations are linked to human diseases [155, 156]. For example, using the CRISPRi approach, Fulco et al. identified 9 distal enhancers and their target genes [155]. CRISPR/Cas9 has also been used to introduce DNA changes in noncoding regions, establishing a link between intronic SNPs in *PHACTR1* and transcriptional function at the locus [94].

Despite the huge progress that has been achieved in our genome editing capability thanks to the CRISPR/Cas9 innovation, some issues remain (Table 1). The therapeutic potential of genome editing in cardiovascular diseases is still restrained by biological and technical problems and it is just starting to be applied. The major limitation with CRISPR/Cas9 is the need of PAM sequences to target and link the nuclease. Two approaches to solve this issue include expanding the number of PAM sequences using sequences from various bacteria and by modifying the PAM sequence specificity of *S. pyogenes* Cas9 [15, 54, 65].

One other major concern for the application of the CRISPR/Cas9 system to animal embryo mutagenesis is related to the off-target effects that have been described at high frequency in human cells. Off-target effects are the consequence of the nonspecific activity of the Cas nuclease in nontarget locations of the genome, due to incorrect binding of the sgRNA [157]. Different groups have evaluated the frequency of off-target events in predicted off-target locations [65, 158]. In one study, editing in the CCR5 and HBB genes, some constructs generated up to a 58% mutation rate in off-target but related CCR2 and HBD DNA sequences in CRISPR/Cas9 transfected cells [159]. However, off-target events have been demonstrated to vary among different cell types [160]. In fact, other studies demonstrated in human iPSCs that this system provides efficient genome-editing tools with high specificity [161–163]. Further, studies in whole organisms show lower off-target frequencies compared to previous studies in cancer cell lines. These studies in mice and monkeys detected no mutations at the predicted off-target sites using CRISPR/Cas9 when generating genome-editing applied to zygotes [29, 31]. Some groups tried to reduce the off-target effects modifying the binding-site of Cas9. Disrupting some binding-sites of Cas9, they were able to cut the DNA on-target, leading to a low or absent off-target binding [54]. Another attempt to reduce off-target effects was the reported use of paired Cas9 nickases instead of Cas9 alone, which significantly diminishes off-target cleavage by 50- to 1000-fold [65]. Even though off-target events might be scarce, they should not be underestimated, since other genes could be mutated with potentially damaging consequences. In particular, it is important for the clinical use of genome-edited cells or tissues to completely avoid the occurrence of off-target effects.

Another issue is the variability of genome-editing efficiency in different tissues, particularly *in vivo*. The CRISPR/Cas9 efficiency seems to be lower in mouse skeletal and cardiac muscles than liver [150]. Even in cardiac-specific Cas9 transgenic mouse, the efficiency of editing is quite low in cardiac cells [30].

A further concern raised for the CRISPR/Cas9 system is related to its editing efficiency: sgRNAs induce Cas9-mediated DSB at the desired target site. DSB stimulates DNA repair through HDR. However, the alternative DNA repair mechanism NHEJ can take place at lower frequencies, introducing unpredictable events of small insertions and deletions [47]. Treatment aimed at promoting HDR have been developed, in order to improve editing efficiency [47].

There are also practical and ethical concerns related to the application of iPSCs engineered and delivered back to the patients. In practice, an efficient and safe delivery of CRISPR/Cas9 into cell types or tissues that are hard to transfect and/or infect and the effect of its introduction into a different genetic background needs to be carefully evaluated [164]. Genetic modifications introduced into embryos or germlines can be transmitted to following generations. Until now, all therapeutic interventions in humans using genome editing has been performed in somatic cells [165, 166], which is ethically accepted, considering the balance between risks and benefits and the use of informed consent. Genome editing in human nonviable embryos has been used to attempt to modify the gene responsible for *β*-thalassaemia by a Chinese group. The efficiency of HDR was low and the edited embryos were mosaic. Moreover, off-target cleavage was also present [167]. The CRISPR-Cas9 system was also used to edit the CCR5 locus in human embryos (related to HIV resistance). Even in the embryos successfully containing the engineered CCR5 allele, it was not possible to control the other alleles at the same locus and they remained wild type or contained in-del mutations [164]. In addition, Ma et al. used the CRISPR/Cas9 system to correct a heterozygous mutation in the *MYBPC3* gene, which can lead to hypertrophic cardiomyopathy, in human embryos specifically created with the sperm of a donor carrying the mutation [168]. In the study, they observed a high number of embryos carrying the wild-type gene without evidence of off-target events. More recently, the CRISPR/Cas9 genome-editing system has been used to investigate the function of the pluripotency transcription factor OCT4 during human embryogenesis. In the study, they specifically targeted the gene encoding OCT4 (*POU5F1*) in diploid human zygotes and found that blastocyst development was compromised, suggesting that OCT4 has an important role in the progression of the human blastocyst [169].

In 2015, the US National Institute of Health (NIH) banned NIH-funded research into genomic editing of human embryos due to serious and unquantifiable safety issues, ethical issues related to the alteration of germ line, which can affect future generations without their consent, and a lack of clear medical applications justifying the use of CRISPR/Cas9 in embryos. However, in June 2015, a Swedish group gained approval for using CRISPR technology to disable genes in human embryos to study embryonic development, and in February 2016, the British Parliament approved the genetic modification of human embryos by using CRISPR/Cas9 and related techniques [170].

## 7. Conclusions

Nowadays, genome editing has become a powerful tool for modifying cell lines and organisms to investigate the biology and the pathophysiological mechanisms of various genetic diseases. The genome editing tools have recently started to be used also in the cardiovascular field to generate new cellular and animal models of cardiovascular diseases. The therapeutic potential of genome editing is still hindered by biological and technical barriers. Another important issue is represented by the ethical concern about the use of CRISPR technology in humans. Progress in the genome editing field will allow us to increase the understanding of the development and pathogenesis of disorders, as well as to attempt to treat cardiovascular diseases.

## Figures and Tables

**Table 1:** Summary of CRISPR advantages and disadvantages.

Advantages	Disadvantages
Target design simplicity	Limited number of PAM sequences
Multiplexed target recognition	Off-site effects
Efficiency for editing different human cells	Mosaicism
Screening DNA noncoding regions	Multiple alleles
	Ethical issues
